# Minocycline declines interleukin-1ß-induced apoptosis and matrix metalloproteinase expression in C28/I2 chondrocyte cells: an in vitro study on osteoarthritis

**DOI:** 10.17179/excli2023-6710

**Published:** 2024-01-24

**Authors:** Amin Moqadami, Mohammad Khalaj-Kondori, Mohammad Ali Hosseinpour Feizi, Behzad Baradaran

**Affiliations:** 1 Department of Animal Biology, Faculty of Natural Sciences, University of Tabriz, Tabriz, Iran; 2 Immunology Research Center, Tabriz University of Medical Sciences, Tabriz, Iran

**Keywords:** osteoarthritis, chondrocytes, minocycline, interleukin-1beta, apoptosis, matrix metalloproteinases

## Abstract

Osteoarthritis (OA) is a degenerative joint disease that occurs with aging. In its late phases, it is determined by the loss of chondrocytes and the breakdown of the extracellular matrix, resulting in pain and functional impairment. Interleukin-1 beta (IL-1β) is increased in the injured joints and contributes to the OA pathobiology by inducing chondrocyte apoptosis and up-regulation of matrix metalloproteinases (MMPs). Here, we aimed to understand whether minocycline could protect chondrocytes against the IL-1β-induced effects. The human C28/I2 chondrocyte cell line was treated with IL-1β or IL-1β plus minocycline. Cell viability/toxicity, cell cycle progression, and apoptosis were assessed with MMT assay and flow cytometry. Expression of apoptotic genes and MMPs were evaluated with qRT-PCR and western blotting. IL-1β showed a significant cytotoxic effect on the C28/I2 chondrocyte cells. The minocycline effective concentration (EC_50_) significantly protected the C28/I2 cells against the IL-1β-induced cytotoxic effect. Besides, minocycline effectively lowered IL-1β-induced sub-G1 cell population increase, indicating the minocycline anti-apoptotic effect. When assessed by real-time PCR and western blotting, the minocycline treatment group showed an elevated level of Bcl-2 and a significant decrease in the mRNA and protein expression of the apoptotic markers Bax and Caspase-3 and Matrix metalloproteinases (MMPs) such as MMP-3 and MMP-13. In conclusion, IL-1β promotes OA by inducing chondrocyte death and MMPs overexpression. Treatment with minocycline reduces these effects and decreases the production of apoptotic factors as well as the MMP-3 and MMP-13. Minocycline might be considered as an anti-IL-1β therapeutic supplement in the treatment of osteoarthritis.

See also the graphical abstract(Fig. 1).

## Introduction

Osteoarthritis (OA) is a degenerative joint disease that develops with age. It is characterized by chondrocyte loss and extracellular matrix degradation in its advanced stages, causing pain and affecting ability among the aging population (Mobasheri, 2013[35]; Ying et al., 2013[51]). Matrix metalloproteinases, free radicals, abnormal cytokine release, estrogen insufficiency, trauma, osteoporosis, and aging have all been associated with OA (Jin et al., 2012[22]; Li et al., 2012[29]; Loeser et al., 2016[32]). Increased articular chondrocyte mortality always occurs in the development of OA cartilage destruction, and its prevention reduces the severity of OA (Heraud et al., 2000[20]; Ryu et al., 2012[39]; Zhang et al., 2014[53]). As a result, chondrocyte apoptosis prevention has slowly emerged as one of the possible alternative treatments for OA (Thomas et al., 2011[47]). 

The extracellular matrix, which is essential to joint function, is synthesized and replaced by chondrocytes - the only cells in cartilage (He and Cheng, 2018[19]). Through increased synthesis of inflammatory mediators and overexpression of matrix metalloproteinases (MMPs), it was proven that the inflammatory cytokines such as interleukin-1β (IL-1β) and TNF-α play important roles in the pathogenesis of OA (Goldring et al., 2011[16]; Wojdasiewicz et al., 2014[49]). Apoptosis of chondrocytes has been demonstrated to be induced explicitly by IL-1β through overexpression of MMPs, particularly collagenases (MMP-3 and -13), which breaks down the extracellular matrix and promotes chondrocyte apoptosis (Aida et al., 2005[1]; Nishitani et al., 2010[37]). According to previous investigations, IL-1β-induced chondrocyte loss results in a substantial increase in caspase-3 activity (Ju et al., 2010[24]). Then, cytochrome c (Cyt c) substantially activates caspase-3 (Antonsson, 2001[2]; Fan et al., 2005[12]). The pro-apoptotic protein Bax, a member of the Bcl-2 family, activates these signaling cascades by sliding through the mitochondrial membrane to promote Cyt c release from mitochondria to cytosol as a result, blocking the mitochondrial route is crucial for preventing cell death and can be accomplished by Bcl-2 by attaching to and suppressing Bax (Suen et al., 2008[41]). In fact, IL-1β is frequently utilized to induce chondrocyte apoptosis and can cause extensive chondrocyte apoptosis (Sanchez et al., 2005[40]). As a result, chondrocytes treated with IL-1β represent an adequate illustration of OA chondrocytes.

Minocycline is a tetracycline antibiotic with immunomodulatory properties that suppresses pro-inflammatory cytokine production and shows anti-apoptotic attributes (Bonelli et al., 2004[3]; Gunn et al., 2020[17]; Metz et al., 2017[34]). Several investigations on minocycline hypothesized mechanisms that strengthen Bcl-2-derived impacts, resulting in protective effects on cells towards apoptosis and suppressing caspase-1 and caspase-3 activation (Wang et al., 2003[48]). Minocycline has also been shown to significantly inhibit interleukin-1-converting enzyme and caspase-3 synthesis, affecting the caspase-dependent pathway in multiple ways and having anti-apoptotic effects (Garrido-Mesa et al., 2013[15]). There is growing evidence that minocycline can suppress MAPK-p38, lower levels of caspase-3-cleaved, and both inducible and endothelial nitric oxide synthase (Bonelli et al., 2004[3]). According to recent investigations, matrix metalloproteinases (MMPs), caspases, and nitric oxide (NO) synthetases, which are all possible targets for the suppression of apoptosis, are all inhibited by minocycline (Elewa et al., 2006[11]; Kim and Suh, 2009[28]). 

In the present study, we treated the C28/I2 chondrocyte cells with IL-1β and investigated the impacts of minocycline on the IL-1β-induced apoptosis and expression of matrix metalloproteinases in the C28/I2 cells.

## Materials and Methods

### Cell lines, reagents, and antibodies

The names and purchased places of materials are as follows: 

Human C28/I2 chondrocyte cell line (Pasteur Institute, Tehran, Iran), Minocycline Hydrochloride (purity>98 %), DMSO (Dimethylsulfoxide), PBS (Phosphate buffer saline), MTT (3-(4,5-Dimethylthiazol-2-yl)-2,5-diphenyltetrazolium bromide), penicillin/streptomycin, Trypsin/EDTA %0.25 and Trypan blue cell staining (Sigma, St. Louis, MO, USA). Recombinant human IL-1β (Purity: >95 %) (Sino Biological, Beijing, China). Primary antibodies against Bax (mouse; cat. no. sc-7480), Bcl-2 (rabbit; cat. no. sc-492), Caspase-3 (mouse; cat. no. sc7272), MMP-3 (mouse; cat. no. sc-21732), and MMP-13 (rabbit; cat. no. sc-30073), β-Actin (mouse; cat. no. sc-47778) all diluted 1:1000 and secondary antibodies against mouse anti-rabbit IgG-HRP (mouse; cat. no. sc-2357) and m-IgGκBP-HRP (mouse; cat. no. sc-516102) all diluted 1:1500 (Santa Cruz Biotechnology, Dallas, TX, USA). RNase A (Simbiolab, Mashhad, Iran). Annexin V-FITC/PI Apoptosis kit (ImmunoStep, Salamanca, Spain). DMEM/F12 (Dulbecco's modified Eagle's medium) (Hyclone, Logan, UT, USA), TRIzol, and FBS (Fetal Bovine Serum) (Gibco) (Thermo Fisher Scientific, CA, USA). SYBR Green RT Master Mix kit (Amplicon, Denmark). ECL chemi-luminescence kit (cat. no. NCI5079) (Thermo Fisher Scientific, Rockford, IL, USA). Phenylmethanesulfonyl fluoride (cat. no. ST505) (Beyotime Institute of Biotechnology, Nanjing, China).

### Cell culturing and treatment

The human C28/I2 chondrocyte cell line was cultured in DMEM/F12 media with 10 % heat-inactivated FBS added at 37 °C in a 90 % humid atmosphere with 5 % CO_2_. To investigate the protective effects of Minocycline (dissolved in ddH_2_O) on IL-1β-induced cytotoxicity, cells were incubated with minocycline for 2 hours and then 10 ng/ml of IL-1β (Du et al., 2015[9]; Rao et al., 2018[38]) was added to the culture, incubated for 24 h to induce the OA cell model. Cells in the control group were cultured untreated. Four groups were examined, including A: Untreated, B: IL-1β, C: Minocycline, and D: Minocycline (2 h prior) + IL-1β. Cell proliferation (duplication time) was detected after incubation for 24, 48, and 72 h, and a trypan blue exclusion test was utilized to assess the proportion of living and dead cells using a hemocytometer.

### Cell viability assay

Human C28/I2 chondrocytes were cultured in T25 flasks and trypsinized after reaching 80 % confluence. Trypsinized cells were centrifuged at 1000 rpm and seeded (5x10^3^/well) in 96-well plates. First, cells were treated with minocycline (5, 10, 15, 20, 25, 30, 35, 40, 45, and 50 µM) for 24, 48, and 72 hours at 37 °C to determine its cytotoxicity in a time-dependent manner. Second, to detect the inhibitory concentration (IC_50_) and effective concentration (EC_50_) of minocycline, cells were treated with minocycline with various concentrations for 24 h at 37 °C. Untreated cells were used as the control group. Third, cells were pre-treated with various concentrations of minocycline for 2 hours before adding IL-1β (10 ng/ml) to determine the protective effect of minocycline against IL-1β cytotoxicity. So, each of the four groups (A, B, C, D) of the cells was treated with 50 μl MTT (2 mg/ml) for 3 h at 37 °C. Purple formazan crystals were dissolved using DMSO (100 μl/well) after incubation. An ELx 800 BioTek microplate reader (San Francisco, CA, USA) was used to measure the absorbance at 570 nm. 

### Apoptosis evaluation

The percentages of chondrocyte apoptosis were assessed by flow cytometry using the Annexin V-FITC/PI Apoptosis kit. 2.5×10^5^ chondrocyte cells per well were cultured in 6-well plates and all four groups were treated for 24 h, re-suspended in 500 μL binding buffer, and stained with 5 μL Annexin V-FITC and 10 µL PI in the dark place at room temperature for 15 min. Following incubation, the samples were examined using a BD FACSCalibur cytometer (Becton Dickinson, San Jose, CA, USA). The apoptotic cell (early and late) percentages were determined using the FlowJo analytic software version 10.

### Cell cycle analysis

C28/I2 cells were plated on a six-well culture plate with a total of 2.5×10^5^ cells per 2 mL and incubated at 37 °C in a CO_2_ incubator for 24 hours. The medium was aspirated and the cells were washed with 1 mL of 1X PBS. Untreated cells were used as control. The cells were treated with IL-1β alone, minocycline (EC_50_), and minocycline (EC_50_) + IL-1β in 2 mL of the culture media. The cells were collected and washed with cold PBS. After being made permeable, the cells were fixed for one hour at 4 °C in extremely chilled 70 % ethanol. The cells were then stained at 37 °C for 15 min using a staining solution including 20 µg/mL RNase A and 50 μg/mL PI in PBS. The BD FACSCalibur cytometer (Becton Dickinson, San Jose, CA, USA) was used to assess the samples.

### RNA extraction, cDNA synthesis, and quantitative real-time PCR (qRT-PCR)

Total RNA from cells was extracted using TRIzol kit. It was quantified with a NanoDrop 2000 UV spectrophotometric analyzer (Thermo Fisher Scientific, CA, USA), and the quality of the RNA samples was assessed using 2 % agarose gel electrophoresis (v/w). PrimeScript RT, Master Mix kit, was used to reverse transcribe RNA into cDNA. The StepOnePlus^TM^ Real-Time PCR System (Applied Biosystems) was used for the qRT-PCR tests. For Bax, Bcl2, Caspase3, MMP3, MMP13, and β-Actin genes (Table 1(Tab. 1)): 10 μl SYBR Green Master Mix, 0.24 μl Reverse primer, 0.24 μl Forward primer, 2 μl cDNA (dilution 1:100), 7.52 μl ddH_2_O in final volume of 20 μl were used. The qRT-PCR protocol consisted of 12 min at 95 °C for primary denaturation, which was continued with 45 cycles at 95 °C for 20 sec, 57 °C for 45 sec, and at 72 °C for 35 sec, and finished with a final extension at 72 °C for 7 min. The 2^-ΔΔCt^ method was used to determine the fold change of each mRNA expression.

### Western blot

Using a lysis buffer (0.1 % SDS, 1 %TritonX‑100, 1.5 M, 1 mM EDTA, 20 mM Tris‑HCl (pH 7.4), 1 % phenylmethanesulfonyl fluoride, and 1.5 M NaCl), the cells were lysed and total protein was purified through a 16,000 g centrifugation at 4 °C for 10 min. Using the DC protein test from Bio-Rad, the protein concentration was calculated. A constant voltage of 80 mV for 30 min, followed by 120 mV for 120 min, was used to separate 30 µg of protein from each group by electrophoresis, which was subsequently transferred to PVDF membranes using the 100 mV for 120 min Bio-Rad TransBlot system (Bio Rad Laboratories, Inc.). 5 % non-fat dried milk dissolved in 10 mM Tris HCl, 0.2 % Tween-20 (TBST), and 0.15 M NaCl were then used to block the membrane for 2 hours at room temperature. The membrane was incubated with the primary antibodies against Bax, Bcl-2, caspase-3, MMP-3, MMP-13, and β-Actin for a whole night at 4 °C. The membrane was incubated with m-IgGκBP-HRP and mouse anti-rabbit IgG-HRP secondary antibodies at room temperature for 2 hours after being rinsed with TBST three times for 5 minutes. The membrane was treated with an ECL chemiluminescence kit and tested with Mini-PROTEAN Tetra Vertical Electrophoresis Cell (Bio-Rad) for analysis. ImageJ v1.8.0 (National Institutes of Health) software was used to densitometry quantification of the images.

### Statistical analysis

GraphPad Prism (San Diego, CA, USA) version 9.0 and SPSS version 26 were used to analyze data. Data normalization was performed as required. Comparison between the two groups was done by a two-tailed t-test and the data were reported as the mean ± SEM. FlowJo v10.8 was used to analyze the data from flow cytometry tests. ImageJ v1.8.0 software was used to perform image analysis and quantify the results obtained from western blotting. Statistical significance was defined as P < 0.05.

## Results

### Cell viability assay and obtaining of the minocycline effective concentration

**Figure 2:**
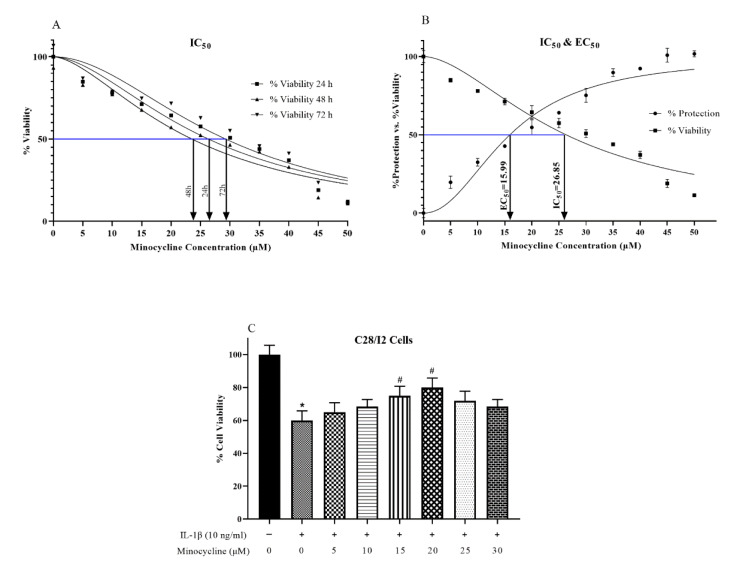
Cell viability assay and effective concentration (EC_50_) of minocycline. (A) Cells were treated with various concentrations of minocycline (0 to 50 µM) for 24, 48, and 72 hours. There was no significant impact based on time. But (B) increasing the concentration of minocycline has detrimental impacts on chondrocyte viability. The 50* %* Inhibitory Concentration (IC_50_) against the C28/I2 chondrocyte cell line is 26.85 µM. Based on these results, the effective concentration (EC_50_) of minocycline has been calculated to be 15.99 µM. (C) Cells were pre-treated with minocycline for 2 h prior to adding 10 ng/ml of IL-1β. It was shown that the percentage of cell viability decreased significantly by adding IL-1β alone compared to the untreated group. However, adding 15 and 20 µM (EC_50_ = 15.99 µM) of minocycline 2 h before adding IL-1β significantly prevented cell loss. *p < 0.05 vs. untreated group and #p < 0.05 vs. IL-1β.

The cell viability was evaluated by MTT test to determine whether minocycline has any potential cytotoxic impact on chondrocytes. Various concentrations of minocycline (5, 10, 15, 20, 25, 30, 35, 40, 45, and 50 µM) were used to treat the cells. Minocycline's cytotoxic impact was assessed 24, 48, and 72 hours following treatment. We observed that minocycline had a concentration-dependent but not time-dependent cytotoxic effect on the chondrocyte cells (Figure 2A(Fig. 2)). Based on these results, subsequent experiments were done at the 24 h time point. On the C28/I2 chondrocyte cell line, IL-1β was used to produce the OA cell model at the suggested concentration of 10 ng/ml. Cells were either incubated for 24 hours with IL-1β (10 ng/ml) and/or pretreated with minocycline (5, 10, 15, 20, 25, 30, 35, 40, 45, and 50 µM) for 2 hours. MTT tests were carried out 24 hours later (Figure 2B, C(Fig. 2)). Results showed that IL-1β significantly decreased cell viability. However, the presence of minocycline at different concentrations significantly reduced the cell damage brought on by IL-1β. Minocycline had the highest protective effect (EC_50_) at 15.99 µM (Figure 2B(Fig. 2)). Therefore, 15.99 µM as the effective concentration of minocycline was used in the subsequent experiments. 

### Minocycline inhibited IL-1β-induced apoptosis 

**Figure 3:**
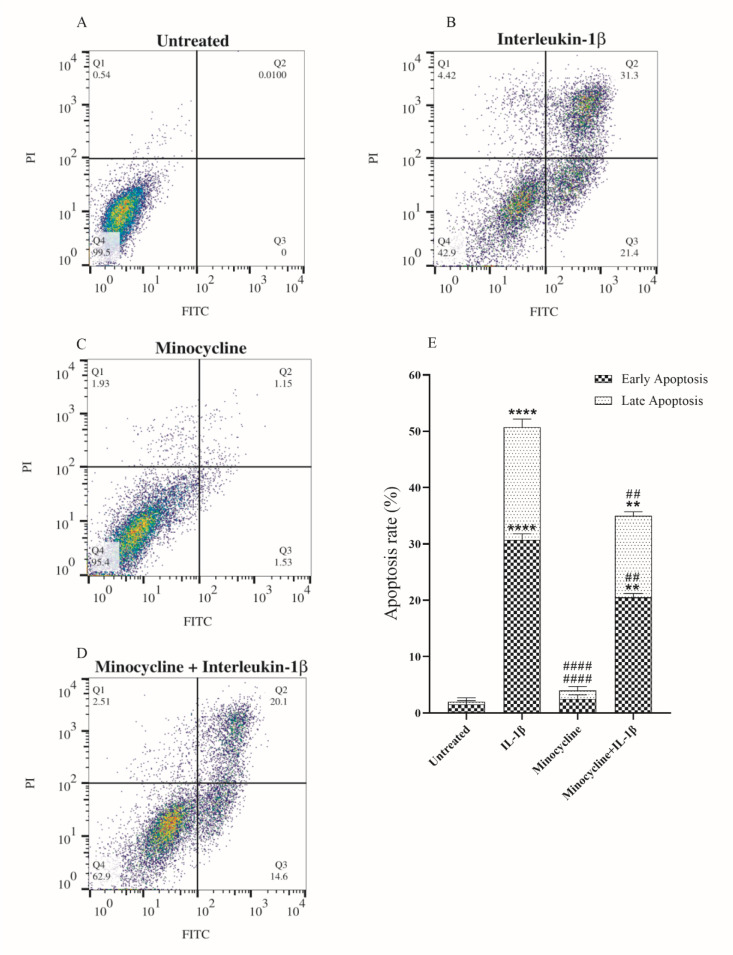
Analysis of cell apoptosis. The effect of minocycline on the apoptosis of the IL-1β-treated C28/I2 chondrocyte cells was measured by flow cytometry. (A) untreated group, (B) C28/I2 cells were treated with 10 ng/mL of IL-1β, (C) cells were treated with EC_50_ concentration of minocycline (15.99 μM), (D) cells were pre-treated with minocycline (15.99 μM) for 2 hours and then IL-1β was added to the cell culture (10 ng/mL) for 24 hours, (E) a graph presenting average percentage of the early and late apoptosis for each treatment of apoptosis assay. Error bars show the SD (standard deviation) of three independent experiments. Significance: **p < 0.01 and ****p < 0.0001 vs. untreated group and ##p < 0.01 and ####p < 0.0001 vs. IL-1β group

To confirm whether the cytotoxicity caused by IL-1β occurs through inducing apoptosis, we assessed apoptosis of the IL-1β-treated C28/I2 cells by flow cytometry. As indicated in Figures 3A and B(Fig. 3), IL-1β significantly induced apoptosis in the C28/I2 cells (21.4 % early and 31.3 % late apoptosis) compared with the untreated group. Prior to adding IL-1β (10 ng/mL), chondrocytes were pretreated with the minocycline (EC_50_ = 15.99 μM) for 2 hours. The results showed a significant reduction in the proportion of apoptotic chondrocytes (14.6 % early and 20.1 % late apoptosis) (Figure 3D(Fig. 3)). Interestingly, treating the cells with the effective concentration of minocycline (EC_50_ = 15.99 μM) caused an insignificant induction of apoptosis in the C28/I2 cells (1.53 % early and 1.15 % late apoptosis) (Figure 3C(Fig. 3)). Figure 3E(Fig. 3) outlines the results obtained from three independent replicates of the experiments. 

### Effect of minocycline on the cell cycle phases of chondrocyte cells 

**Figure 4:**
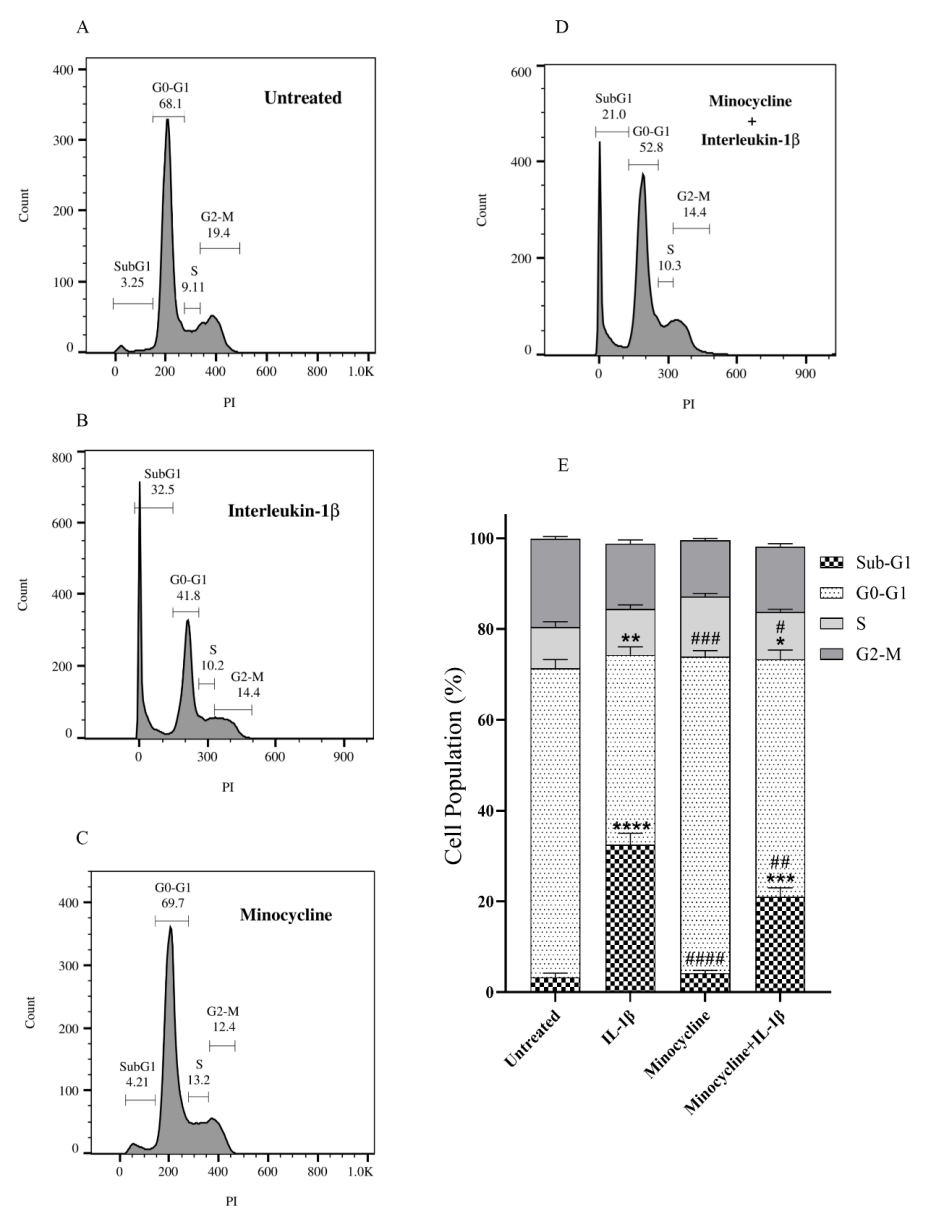
Cell cycle phase distribution analysis of untreated and treated C28/I2 chondrocyte cells. (A) Cell cycle phase distribution of untreated cells. (B) After being treated with a 10 ng/ml concentration of IL-1β, there is a significant increase in the number of cells in the sub-G1 phase, indicating the apoptotic cells. (C) Cells are treated with EC_50_ concentration of minocycline, and there is no significant change in the cell cycle phases compared to the untreated cells. (D) Cells were treated with 10 ng/ml IL-1β + 15.99 µM minocycline for 24 hours, and the results showed a significant decrease in apoptotic cells compared to the IL-1β alone group. (E) Outline of the results obtained from three independent cell cycle phase distribution assays. Error bars show the SD (standard deviation) of three separate experiments. Significance: *p < 0.05, **p < 0.01, ***p < 0.001, ****p < 0.0001 vs. untreated and #p < 0.05, ##p < 0.01, ###p < 0.001 and ####p < 0.0001 vs. IL-1β.

Genomic DNA fragments into smaller fragments, each 180 base pairs (bp) or multiples of 180 bp, during apoptosis. Since this is a particular indicator of apoptosis, flow cytometry could be used to quantify apoptosis. For flow cytometry, cells are treated with Propidium Iodide (PI). While PI is excluded by the viable cells, it can enter the apoptotic cells and stain their fragmented genome, resulting in the Sub-G1 peak, which appears below the G1 peak and signifies the induction of apoptosis (Haridevamuthu et al., 2023[18]). Therefore, a flow cytometry assay was conducted to determine the effects of minocycline, IL-1β, and minocycline+IL-1β treatments on the cell cycle progression of C28/I2 chondrocyte cells. Minocycline effective concentration treatment showed an insignificant change in the sub-G1 cell phase population (4.21 %) compared to the untreated cells (3.25 %) (Figure 4A and C(Fig. 4)). On the other hand, treatment with IL-1β significantly increased the sub-G1 cell phase population (32.5 %) compared to the untreated control cells (3.25 %). Interestingly, two hours of pretreatment with the effective concentration of minocycline (in the minocycline+IL-1β treatment group) significantly decreased the sub-G1 cell phase population (21 %) compared to the IL-1β treated cells (32.5 %) (Figure 4B and D(Fig. 4)). Figure 4E(Fig. 4) outlines the results obtained from three independent assays. 

### Effect of minocycline on the expression of apoptotic genes 

**Figure 5:**
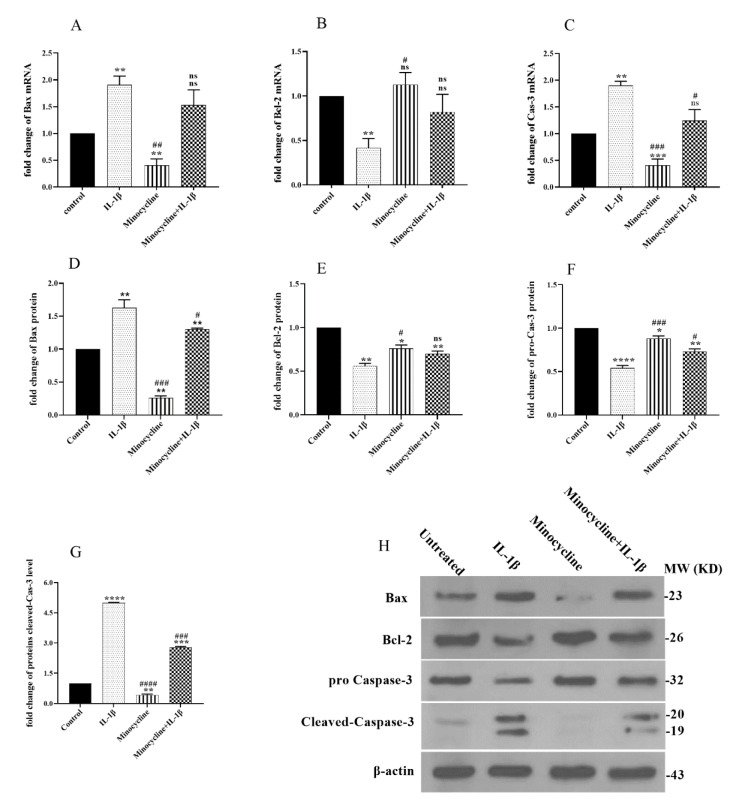
Protein and mRNA expression analysis of the apoptotic genes. The cells were pretreated with minocycline (EC_50_ = 15.99 µM) for 2 hours before subsequent treatment with 10 ng/ml IL-1β for 24 hours. The mRNA expression levels of (A) Bax, (B) Bcl-2, and (C) Caspase-3 were assessed by qRT-PCR, and the protein expression levels of (D) Bax, (E) Bcl-2, (F) pro-Caspase-3, and (G) cleaved Caspase-3 were measured by western blot, respectively. (H) Selected blots reflecting corresponding protein levels. Data are shown as mean ± SEM. All experiments were examined three times. Significance: ns > 0.05, *p < 0.05, **p < 0.01, ***p < 0.001, ****p < 0.0001 vs. untreated and ns > 0.05 #p < 0.05, ##p < 0.01, ###p < 0.001 and ####p < 0.0001 vs. IL-1β.

The mRNA and protein expression levels of Bax, Bcl-2, and Caspase-3 were assessed by qRT-PCR and western blotting to ascertain whether the mitochondrial apoptotic pathway was impacted. As shown in Figure 5A-E(Fig. 5), IL-1β treatment led to a significant decrease in the mRNA and protein levels of Bcl-2 (anti-apoptotic) and a significant increase in the mRNA and protein levels of Bax and Caspase-3 (pro-apoptotic). However, minocycline treatment significantly increased the expression of anti-apoptotic Bcl-2 while significantly decreased the expression of pro-apoptotic Bax and Caspase-3 genes. Western blotting confirmed these effects at the protein level (Figure 5A-H(Fig. 5)). 

### Effects of minocycline on the cartilage degrading collagenases MMP-3 and MMP-13 

**Figure 6:**
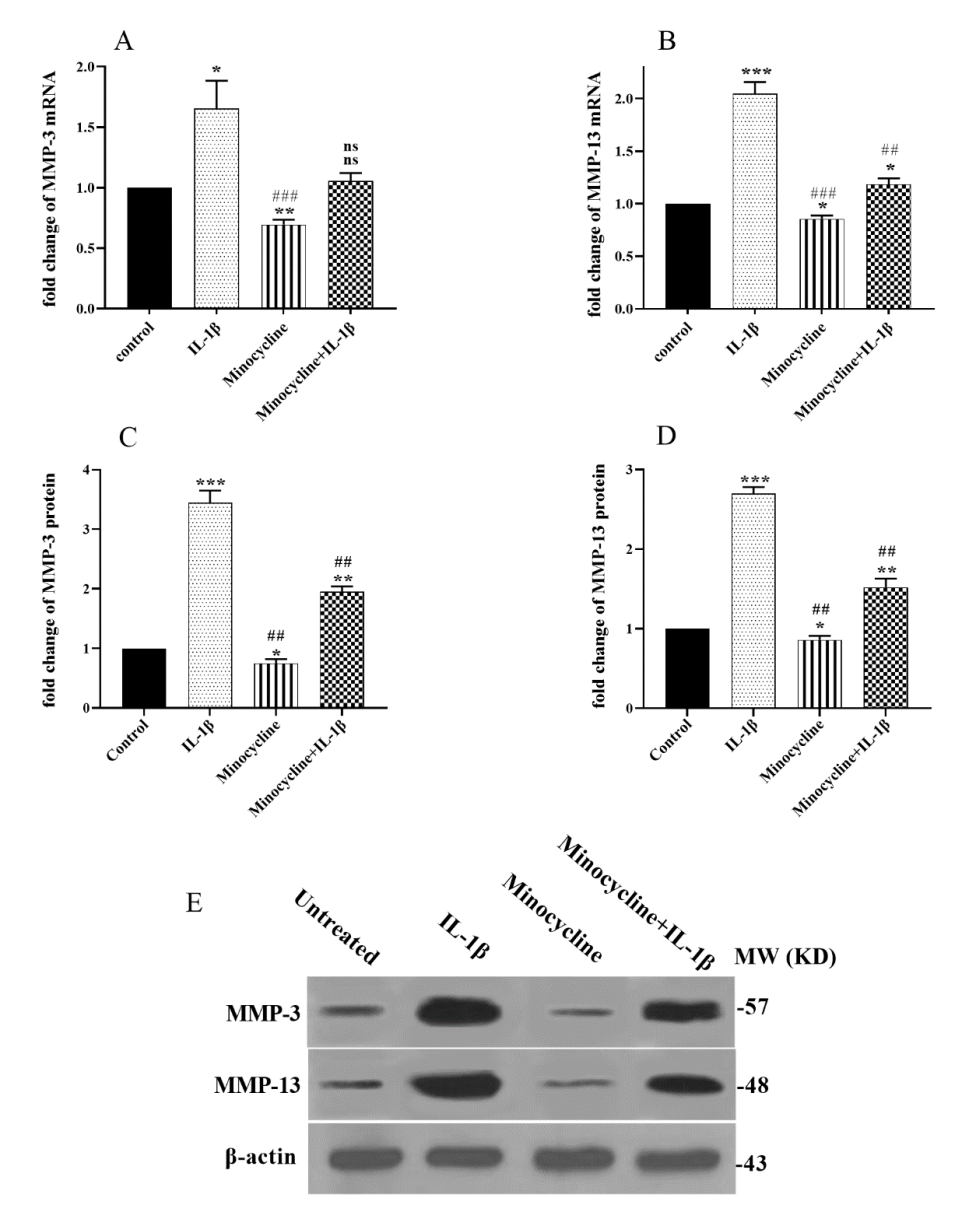
Effect of minocycline on the cartilage degrading collagenases. The mRNA expression level of (A) MMP-3 and (B) MMP-13 collagenases were assessed via qRT-PCR. And also protein levels of (C) MMP-3 and (D) MMP-13 were evaluated by western blot. (E) Selected blots reflecting corresponding protein levels. Therefore, minocycline reduced their expression and protected the cartilage matrix from IL-1β-induced apoptotic degradation. Data are shown as mean ± SEM. All experiments were examined three times. Significance: ns > 0.05, *p < 0.05, **p < 0.01, and ***p < 0.001 vs. untreated and ns > 0.05 #p < 0.05, ##p < 0.01 and, ###p < 0.001 vs. IL-1β.

Expression of collagenases MMP-3 and MMP-13, which accelerate chondrocyte apoptosis and eliminate native collagen fibers, is induced by IL-1β (Aida et al., 2005[1]; Rao et al., 2018[38]). MMP-3 and MMP-13 play crucial roles in cartilage destruction. To understand whether minocycline can affect the expression of these cartilage-degrading enzymes, we evaluated their expression in the OA cell model by qRT-PCR. As depicted in Figure 6(Fig. 6), treatments with the IL-1β significantly increased MMP-3 and MMP-13 expressions both at the mRNA and protein levels in the OA model cells. Fascinatingly, minocycline treatment significantly decreased the IL-1β-induced MMP-3 and MMP-13 collagenases expression (Figure 6A-E(Fig. 6)).

## Discussion

In osteoarthritis, chondrocytes are lost, and the extracellular matrix is degraded, leading to pain and limited function of joints. Although medicines and surgery are used to treat OA, no satisfactory results have been obtained. OA patients had considerably higher rates of chondrocyte apoptosis but significantly lower rates of cell survival (Huang et al., 2016[21]). It has been demonstrated that healthy chondrocytes are required to preserve extracellular matrix stability, and chondrocyte death promotes OA progression and articular cartilage deterioration (Kim and Blanco, 2007[26]; Thomas et al., 2007[46]). So, strategies to reduce chondrocyte apoptosis and enhance cell survival might be effective alternative treatments for OA. It was reported that the inflammatory cytokine IL-1β is increased in the synovial fluid of individuals with OA and triggers the chondrocyte apoptosis, as a result, may be contributed to the pathophysiology of OA (Liu et al., 2006[31]; Massicotte et al., 2002[33]; Theoleyre et al., 2004[45]; Yu et al., 2023[52]). Accordingly, anti-IL-1β therapy is now considered as an effective therapeutic option for OA patients (Kapoor et al., 2011[25]; Rao et al., 2018[38]). However, as concluded in a recent systematic review and meta-analysis, anti-IL-1β therapy is possibly associated with an increased risk of adverse events (Yu et al., 2023[52]). Infections, gastrointestinal disorders, skin disorders, neutropenia, injection site reactions, headache, respiratory system disorders, and diarrhea are frequent adverse effects in OA patients treated with different anti-IL-1 therapeutics (Yu et al., 2023[52]). Furthermore, serious adverse effects, including hemorrhagic diarrhea, pneumonia, Staphylococcus infection, and pancreatitis, were reported in OA patients treated with IL-1 receptor antagonists (Yu et al., 2023[52]).

On the other hand, minocycline has been found to prevent cell death by two putative mechanisms: 1) reducing both adaptive and innate immunity and 2) blocking apoptotic cascades, leading to an overall anti-inflammatory impact and protecting cells from death (Fernandes, 2022[13]). We hypothesized that minocycline could alleviate IL-1β induced effects in the OA patients. To test this hypothesis, we conducted an *in vitro* OA cell model study by treating the C28/I2 chondrocyte cells with IL-1β. As depicted in Figure 3(Fig. 3), we demonstrated that IL-1β efficiently induced apoptosis of the C28/I2 chondrocyte cells. Besides, when the C28/I2 cells were pre-/co-treated with the EC_50_ concentration of minocycline, the count of IL-1β-induced apoptotic cells was significantly decreased. This observation implied that minocycline could effectively preserve chondrocyte cells against IL-1β-induced apoptosis. Notably, the EC_50_ concentration of minocycline showed a very low and ignorable apoptotic effect on the cells, highlighting its safety on the chondrocyte cells. We confirmed these observations with the cell cycle phase analysis. As shown in Figure 4(Fig. 4), minocycline efficiently reduced the ratio of the Sub-G1 cell population, which was significantly increased in the IL-1β treated cells. 

Anti-IL-1β effects of minocycline were further confirmed by the expression analysis of the pro-apoptotic Bax, caspase-3, and anti-apoptotic Bcl-2 factors. Figure 5(Fig. 5) shows that minocycline significantly reduced the IL-1β-induced Bax and caspase-3 and increased Bcl-2 expression levels both at the mRNA and protein levels. Our findings are in line with the previous report by Chen et al., who demonstrated that minocycline could inhibit caspase-3 and caspase-1 and postpone mortality in a transgenic mouse model of Huntington disease (Chen et al., 2000[4]). Besides, our findings confirmed the enhancement of Bcl-2-derived effects thereby preventing from apoptosis by minocycline, which were already reported by others (Domercq and Matute, 2004[8]; Jordan et al., 2007[23]; Wang et al., 2003[48]). Members of the Bcl-2 family are well recognized for modulating chondrocyte survival and apoptosis, as well as activating the caspases cascade (Nishitani et al., 2006[36]; Sun et al., 2002[42]). Bcl-2 levels in OA cartilage are lower than in normal cartilage, suggesting decreased chondrocyte survival (Kim et al., 2000[27]). 

We also evaluated the effect of minocycline on the matrix metalloproteinases' expression (MMPs) in the C28/I2 cells because MMPs de-regulation contributes to the OA pathobiology. Chevalier reported that IL-1 promotes MMP production and declines the synthesis of proteoglycans and type II collagen (Chevalier, 1997[5]). Besides, higher expression of IL-1β is associated with the production of the MMPs (Dunn et al., 2014[10]). MMPs are the major enzymes involving in the degradation of the main components of the extracellular matrix of cartilage, such as proteoglycans and type II collagen (Li et al., 2017[30]). Previous studies have reported that expression of MMP-1, MMP-3, and MMP-13 are increased in the OA cartilage (Davidson et al., 2006[7]; Tetlow et al., 2001[44]). The increased MMP synthesis in injured chondrocytes led to an imbalance in the proteoglycan secretion and breakdown, severely reducing cartilage's ability to support bone (Li et al., 2017[30]). Besides, the upregulation of MMP-1, MMP-3, and MMP-13 promotes type II collagen and aggrecan degradation, which are the main components of articular cartilage (Fernandes et al., 2002[14]). Consistent with other studies (Tang and Dong, 2017[43]; Yao et al., 2017[50]), when we treated the C28/I2 chondrocyte cells with IL-1β, it led to significantly elevated expression levels of MMP-3 and MMP-13. As depicted in Figure 6(Fig. 6), minocycline treatment significantly reduced the expression of these genes in both mRNA and protein levels. These results confirm previous reports that minocycline could inhibit various MMPs, both *in vitro *and* in vivo*, preventing pathogenic tissue destruction (Fernandes, 2022[13]).

Based on the literature, although non-significant but, the main and considerable adverse effects of the anti-IL-1β therapy against OA are infectious diseases in the respiratory and gastrointestinal systems, including diarrhea, pneumonia, Staphylococcus infection, and pancreatitis (Chevalier et al., 2009[6]; Yu et al., 2023[52]). Alongside the anti-inflammatory, anti-apoptotic, and anti-MMPs effects, minocycline is primarily a well-known antibiotic drug (Bonelli et al., 2004[3]; Gunn et al., 2020[17]; Metz et al., 2017[34]). As a result, supplementing it during anti-IL-1β therapy of OA patients might be more helpful in combating the probable infections. 

In conclusion, this study confirmed that IL-1β causes chondrocyte apoptosis and MMPs upregulation, which promote cartilage deterioration. We showed that minocycline treatment significantly ameliorates IL-1β-related effects on the C28/I2 chondrocyte cells and declines expressions of the MMP-3, MMP-13, and apoptotic factors. These findings highlighted that minocycline could be considered not only as a potential treatment for OA but also as a supplement during the anti-IL-1β therapy of the OA patients.

## Declaration

### Acknowledgment

This study was supported by the research deputy of the University of Tabriz.

### Conflict of interest

The authors declare that they have no conflict of interest. 

### Funding

No funds, grants, or other supports were received.

## Figures and Tables

**Table 1:**
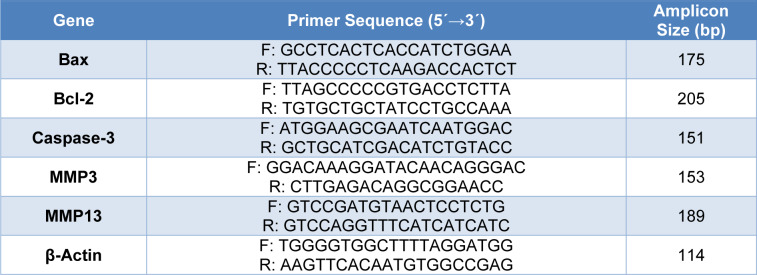
The primer pairs were used for qRT-PCR

**Figure 1:**
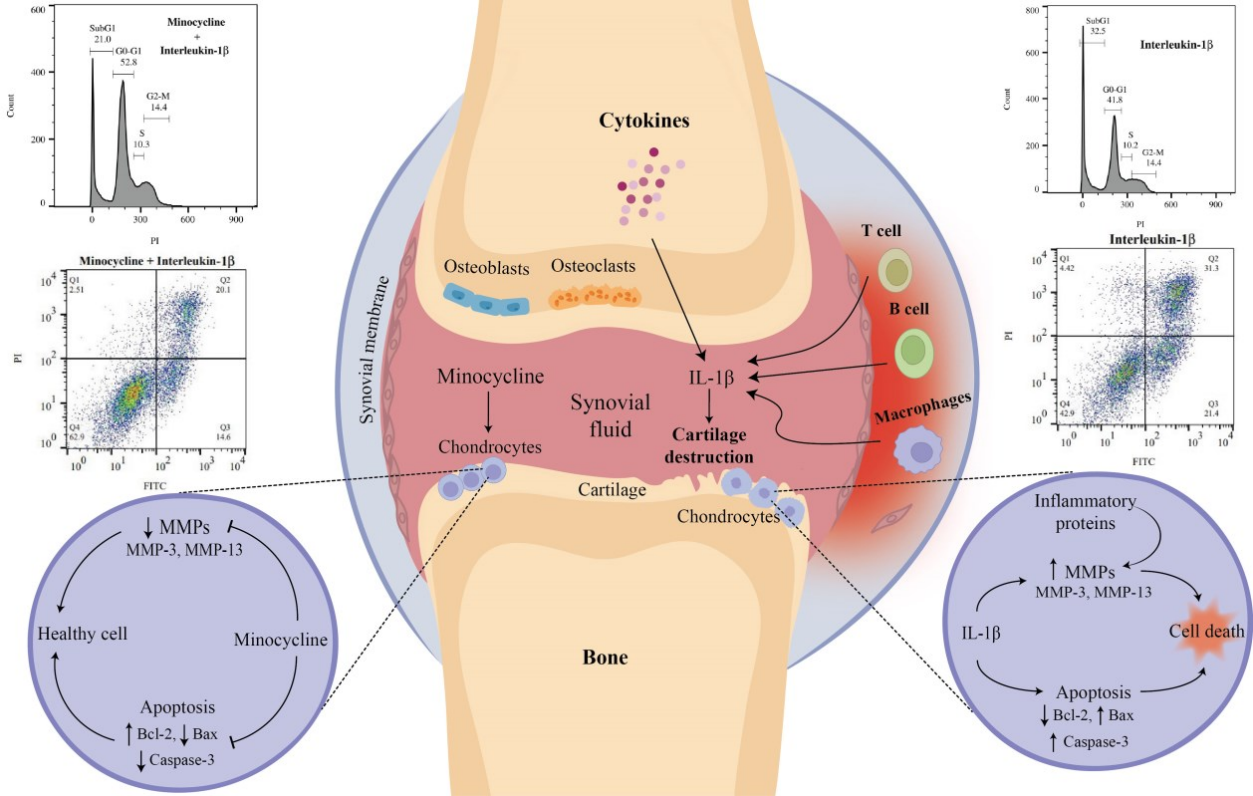
Graphical abstract. Osteoarthritis is an age-related degenerative joint disease and interleukin-1beta is one of the main cytokines which causes it. In this study we investigated the protective effects of minocycline to reduce interleukin-1beta-induced apoptosis by preventing apoptotic genes and also matrix metalloproteinases expression. These findings highlighted that minocycline could be considered not only as a potential treatment for OA but also as a supplement during the anti-IL-1β therapy of the OA patients.
